# Swimming emissions from dogs treated with spot‐on fipronil or imidacloprid: Assessing the environmental risk

**DOI:** 10.1002/vetr.5560

**Published:** 2025-05-23

**Authors:** Rosemary Perkins, Gaëtan Glauser, Dave Goulson

**Affiliations:** ^1^ School of Life Sciences University of Sussex Brighton UK; ^2^ Neuchâtel Platform of Analytical Chemistry University of Neuchâtel Neuchâtel Switzerland

## Abstract

**Background:**

Fipronil and imidacloprid are increasingly recognised as contaminants of concern in aquatic environments. This study aimed to quantify swimming emissions from dogs treated with spot‐on fipronil or imidacloprid, assess the associated environmental risks and evaluate whether current label instructions on swimming restrictions are adequate.

**Methods:**

Emissions from swimming were measured for 49 dogs treated with spot‐on fipronil or imidacloprid on days 5, 14 or 28 post‐application. The environmental risk was assessed by calculating risk quotients, dividing the predicted environmental concentrations by the predicted no‐effect concentrations for freshwater ecosystems.

**Results:**

Mean washoff ranged from 4% to 0.4% of the applied dose for fipronil and 10% to 1.4% for imidacloprid across the 5–28‐day period. Risk quotients indicate a risk to aquatic ecosystems throughout the products' duration of action.

**Limitations:**

The results may underestimate emissions for fipronil, as swimming is permitted from 3 days post‐application and measurements began on day 5.

**Conclusion:**

This study highlights clear ecological risks from spot‐on parasiticides and provides evidence that current label instructions on swimming do not provide sufficient environmental protection. Risk‐based parasite control strategies and extended swimming restrictions are recommended. Regulatory review of environmental risk assessments and mitigation measures is warranted to protect aquatic environments.

## INTRODUCTION

Fipronil and imidacloprid are potent insecticides widely used for the treatment and prevention of fleas on cats and dogs and are typically applied as monthly spot‐on treatments.^1^ Originally developed for crop protection in the late 1980s and early 1990s,^2^, ^3^ their agricultural use is now heavily restricted in the European Union (EU) and UK due to environmental concerns, particularly regarding their adverse effects on pollinators.^4^, ^5^ As persistent, mobile and toxic (PMT) chemicals, they are increasingly recognised as emerging contaminants of concern for aquatic ecosystems, and potentially for human health,^6^, ^7^, ^8^ with several studies demonstrating the degradation of aquatic communities associated with exposure to these compounds.^8^, ^9^, ^10^, ^11^, ^12^

Recent reports reveal widespread fipronil and imidacloprid contamination of fresh waters in the UK despite restrictions on agricultural use, frequently occurring at concentrations that ecotoxicity studies have shown can harm aquatic life.^13^, ^14^, ^15^ These findings have prompted scrutiny of the role of pet parasiticides in the measured pollution and of the regulatory framework governing their use.^16^ New research has shown that ‘down‐the‐drain’ household transfer from treated pets, and subsequent entry via wastewater, is a major source of fipronil and imidacloprid freshwater pollution.^17^ High concentrations and strong positive correlations with dog swimming activity have also been demonstrated in dog swimming ponds, indicating that dog swimming is a further source of surface water pollution.^18^ However, little is known about swimming emissions from individual treated dogs, or the overall contribution of this pathway to surface water pollution, to help inform responsible prescribing practices and evidence‐based mitigation strategies.

Under the International Cooperation on Harmonisation of Technical Requirements for Registration of Veterinary Medicine Products (VICH) framework, environmental risk assessments for veterinary medicines used in non‐food animals assume negligible environmental exposure. Consequently, no phase II risk assessment, requiring emissions and ecotoxicity data, is typically required prior to regulatory approval.^19^ In light of recent research indicating widespread freshwater pollution from pet parasiticides, regulators in the EU and UK have concluded that the current approach should be reviewed.^20^, ^21^

In 2011, the European Medicines Agency published a reflection paper on risk mitigation measures for veterinary medicinal products, recognising the potential for emissions via dog swimming. As a precaution, the paper recommended a minimum 48‐hour interval between topical application of parasiticides and access to waterbodies for treated dogs. However, this default interval is not based on product‐specific scientific data.^21^

A targeted phase II environmental risk assessment for dog swimming was conducted for Advocate Spot‐on Solution (Elanco, containing moxidectin and imidacloprid). This assessment calculated a predicted environmental concentration (PEC), based on the assumption that the full dose for the maximum size pipette enters a waterbody of 100 m^3^, and concluded that imidacloprid poses no significant risk to aquatic species. However, a 4‐day swimming restriction was advised to mitigate risks associated with moxidectin.^22^ Notably, this assessment used a safe environmental threshold, or predicted no‐effect concentration (PNEC), of 850,000 ng/L for imidacloprid—several orders of magnitude higher than current PNEC estimates.^23^, ^24^ The original PNEC was derived from ecotoxicity studies on the water flea *Daphnia magna*,^25^ a test species now known to exhibit unusually high tolerance to imidacloprid.^26^, ^27^ Further detail on the methodology used to derive this PNEC is provided in the Supporting Information.

In this study, we hypothesise that fipronil and imidacloprid swimming emissions from spot‐on treated dogs pose a risk to aquatic ecosystems throughout the product's duration of action and that label instructions on minimum waiting intervals before swimming do not provide adequate environmental protection. To test this hypothesis, we measured fipronil and imidacloprid swimming emissions from spot‐on treated dogs on days 5, 14 and 28 post‐application and assessed the environmental risk posed by these emissions through the calculation of risk quotients.

## METHODS

### Sample collection

Forty‐nine dogs were volunteered by their owners; only healthy dogs that had not been treated with a parasiticide product containing fipronil or imidacloprid within the preceding 3 months were eligible for inclusion. All the animals received a general health and weight check from a veterinarian prior to spot‐on application. Twenty‐five dogs were treated with Frontline Spot‐on Dog 10% (w/v) (Boehringer Ingelheim, containing 67–402 mg fipronil, depending on the size of dog to be treated) and 24 dogs were treated with Advocate Spot‐on Solution (Elanco, containing 40–400 mg imidacloprid and moxidectin), following the manufacturers’ guidelines on dosage and application (see Supporting Information). After treatment, dogs returned on days 5, 14 or 28 post‐application for sample collection. Dogs were placed in a plastic tub of water at approximately mid‐shoulder depth (range 37–414 L) at a temperature of 20°C (within the normal range for inland water in the UK^26^) for approximately 5 minutes to simulate swimming. All samples were analysed for both imidacloprid and fipronil controls—namely, imidacloprid‐treated dogs were controls for fipronil‐treated dogs and vice versa. Following swimming, the volume of water in the tub was measured using a combination of pre‐applied calibration markings on the collecting tub (measured to the litre) and a volumetric 1 L beaker (measured to the nearest 100 mL).

Samples were collected in 15 mL lightsafe polypropylene Falcon tubes and stored at ‒20°C prior to being sent by express air shipment (also frozen) to Neuchâtel Platform of Analytical Chemistry, University of Neuchâtel, Switzerland, for analysis. The tub was cleaned with detergent and ethanol wipes between sampling events, and four replicate equipment blanks were taken. The extraction and analysis of imidacloprid and fipronil were performed according to Perkins et al.^17^ Detailed information on the methodology is available in the Supporting Information.

Statistical analyses to assess the effect of time on washoff were performed using Box‒Cox transformed linear regression. All analyses were performed and all figures were created using R Studio software (version 1.2.5042‐1).

### Calculation of emissions through swimming and environmental risk assessment

Emissions were calculated as mass washoff and washoff percentage per dog based on measured concentrations of active ingredient (AI), where:

(1) $$\begin{array}{ccc} \;\;\;\;\;\;\;\mathrm{Mass}\;\mathrm{washoff}\;\left(\mathrm{mg}\right) & = & \mathrm{Rinsate}\;\mathrm{volume}\;\left(L\right)\\ & & \times \;\mathrm{AI}\;\mathrm{concentration}\;\left(\mathrm{mg}/L\right)\; \end{array}$$

(2) $$\mathrm{Washoff}\;\mathrm{percentage}=\frac{100\;\times \;\mathrm{mass}\;\mathrm{washoff}\;\left(\mathrm{mg}\right)}{\mathrm{mass}\;\mathrm{applied}\;\mathrm{per}\;\mathrm{dog}\;\left(\mathrm{mg}\right)}\;$$

To assess the environmental risk posed by treated dogs entering waterbodies, PECs were calculated based on mean washoff percentages at days 5, 14 and 28 post‐application, for a 45 kg dog treated with 400 mg imidacloprid or 534 mg fipronil (Tables S1 and S2) entering a 100 m^3^ (10 m × 10 m × 1 m deep, 100,000 L) waterbody, based on the PEC calculation in the phase II assessment performed for Advocate.^22^ Based on previous related studies and risk assessments, the assumption was made that the AI dispersed evenly throughout the waterbody and the entire AI dose was administered.^17^, ^22^

Risk was assessed using risk quotients, which were calculated by dividing the PEC by the PNEC. The PNEC is the maximum concentration of a chemical in the environment below which no harmful effects are expected in an ecosystem. PNECs are derived using various methods, with the choice depending on the availability and quality of ecotoxicity data. The most widely used approach is the assessment factor method, which applies assessment factors to ecotoxicological data from the most sensitive species. These factors (typically between 1 and 100) help extrapolate findings from laboratory tests, single‐species studies and short‐term experiments to predict real‐world ecosystem effects. Given that PNECs reflect the current state of knowledge, they should be reviewed regularly.^28^, ^29^

As PNECs can vary depending on their derivation and source, a range were used here: 6.8 ng/L and 0.77 ng/L for imidacloprid and fipronil, respectively, representing the lowest PNEC in freshwater sourced from the NORMAN Association in December 2024,^25^ as well as PNECs of 4.8 ng/L for imidacloprid and 12.1 ng/L for fipronil, from European Chemicals Agency biocide assessments.^23^, ^30^ The methods used to derive the PNECs included in this analysis are described in the Supporting Information.

Risk quotients were interpreted in line with the phase II environmental risk assessment for veterinary medicines, with less than 1 indicating no risk and greater than 1 indicating a risk.^31^

To assess total emissions from the dog population through swimming and compare this to down‐the‐drain emissions described elsewhere,^17^ population emission fractions (PEFs), defined as the percentage of total applied AI emitted via a defined pathway from a population, were estimated by modelling swimming emission results together with survey data on the reported frequency of swimming.^32^ Estimation of the PEF was performed according to Perkins et al.^17^ Detailed information on the methodology is available in Table S3.

## RESULTS

### Swimming emissions

**FIGURE 1:**
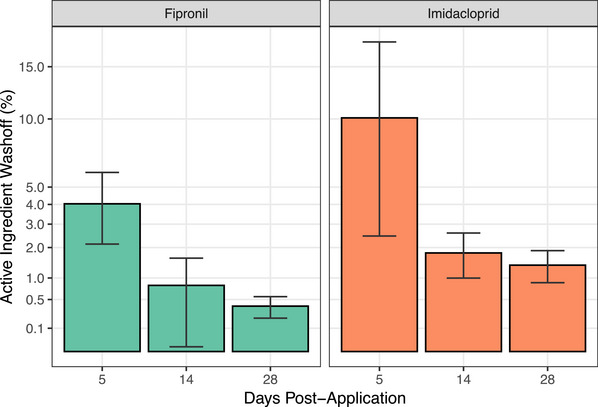
Mean washoff percentages for fipronil and imidacloprid from spot‐on treated dogs on days 5, 14 and 28 post‐application through swimming, displayed as a percentage of the applied mass. Individual dogs were only washed on one occasion (i.e., washes were not sequential). Error bars indicate 95% confidence intervals. The *Y*‐axis is square root transformed.

Fipronil and imidacloprid were detected in 100% of swimming samples from treated dogs, with mean washoff percentages of 4.0%, 0.8% and 0.4% for fipronil and 10.1%, 1.8% and 1.4% for imidacloprid on days 5, 14 and 28 post‐application (Figure 1 and Table S4). Washoff decreased over time post‐application (*p* < 0.01).

Imidacloprid and fipronil were detected in three of 25 and two of 24 of the swimming samples from reciprocal control dogs, respectively. The average mass washoff from untreated control dogs was less than 2% of that observed in treated dogs for both compounds (Table S4), indicating that inadvertent contamination was unlikely to have influenced the study results. Neither compound was detected in equipment blanks.

### Predicted environmental concentrations

**FIGURE 2:**
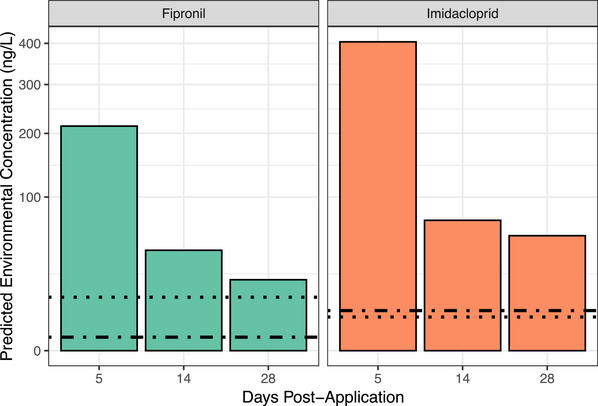
Predicted environmental concentrations for a 45 kg dog treated with spot‐on fipronil or imidacloprid entering a 100 m^3^ waterbody at 5, 14 and 28 days post‐application, based on mean washoff percentages in this study. Square root transformed *Y*‐axis. Dotted lines indicate predicted no‐effect concentrations (safe thresholds) from European Chemicals Agency biocide assessments of 12.1 ng/L for fipronil and 4.8 ng/L for imidacloprid,^23^, ^30^ and dashed lines indicate predicted no‐effect concentrations from the NORMAN Association of 0.77 ng/L for fipronil and 6.8 ng/L for imidacloprid.^24^

PECs and risk quotients are provided in Figure 2 and Table 1. PNECs were exceeded for both compounds following a single swimming event for at least 28 days following application (Figure 2).

**TABLE 1:** Predicted environmental concentrations (PEC) and risk quotients for a 45 kg dog treated with spot‐on fipronil or imidacloprid entering a 100 m^3^ waterbody 5, 14 and 28 days post‐application, based on mean washoff percentages in this study.

	Day	PEC (ng/L)	Risk quotients based on PNECs	
Imidacloprid			ECHA (4.8 ng/L)	NORMAN (6.8 ng/L)
	5	404.0	84.2	59.4
	14	72.0	15.0	10.6
	28	56.0	11.7	8.2
Fipronil			ECHA (12.1 ng/L)	NORMAN (0.77 ng/L)
	5	213.6	17.6	277.4
	14	42.7	3.5	55.5
	28	21.3	1.7	27.7

*Note*: Risk quotients based on predicted no‐effect concentrations (PNECs) from European Chemicals Agency (ECHA) biocides assessments^23^, ^30^ and NORMAN database.^24^

### Population emissions

Based on reported swimming frequency, population emission fractions (the proportion of total AI applied to the population emitted through swimming) were estimated at 0.8% and 1.4% for fipronil and imidacloprid, respectively. Total emissions through swimming were therefore lower than those occurring down‐the‐drain, which previous research has estimated to account for 6.0% of fipronil and 9.1% of imidacloprid applied in spot‐on treatments to dogs.^17^

## DISCUSSION

These results show that swimming emissions from dogs treated with spot‐on fipronil or imidacloprid occur for at least 28 days after application, posing a risk to aquatic ecosystems throughout this period. These findings indicate that current datasheet guidelines, which permit swimming from 3 days after application of spot‐ons containing fipronil and 5 days after spot‐ons containing imidacloprid and moxidectin,^33^ do not provide adequate environmental protection. Instead, the evidence suggests that treated dogs should not be allowed to enter waterbodies for the licensed duration of action of these products (4 weeks) to minimise the risk to aquatic ecosystems.

While the data indicate that overall emissions through swimming are lower than those occurring via down‐the‐drain pathways (e.g., bathing, bed washing and owner handwashing), localised impacts may still occur. Indeed, with spot‐on treated dogs entering UK waterbodies more than 3 million times every year,^32^ there is considerable potential for direct parasiticide emissions through swimming, particularly in standing waterbodies entered by multiple dogs.

There are several potential strategies to effectively reduce swimming emissions from dogs treated with spot‐on products. Previous research found that 86% of dog owners were unaware of the environmental risks associated with pet parasiticides, indicating considerable scope for improved product labelling and consumer awareness.^18^ Transitioning from routine year‐round prophylaxis of small animal ectoparasites towards a risk‐based approach that targets higher risk animals or confirmed infestations—as recommended by multiple British veterinary associations^34^—would both reduce parasiticide emissions and help to mitigate other potential adverse consequences of overuse, such as resistance.^35^ It should be noted that little is known about the environmental emissions and risks associated with alternative ectoparasiticide products, including the newer generation of systemic isoxazolines; however, the available evidence—including demonstration of fluralaner in swimming water following entry of an orally treated dog—indicates potential concerns.^36^, ^37^ Sales data indicating a shift towards isoxazolines in recent years^32^, ^36^ highlight the urgent need for further research on the fate and impact of other pet parasiticides to avoid the occurrence of ‘regrettable substitution’, where hazardous products are replaced with alternatives that pose similar or greater risks.

Regulatory measures to mitigate fipronil and imidacloprid emissions could include advising extended swimming restrictions for spot‐on treated dogs and restricting product availability, as many spot‐on products containing fipronil and imidacloprid are available for general sales in the UK, with no requirement for a prescription from a veterinarian or other suitably qualified person.^38^

Scientific understanding of the environmental and human health risks posed by novel chemicals is constantly evolving. Estimates of safe environmental thresholds can vary significantly based on the representation of sensitive species in datasets and the assessment factors applied. For newly introduced chemicals, aquatic ecotoxicity data are often limited to a few species, whereas river ecosystems may support hundreds.^39^ This creates uncertainty, potentially leading to either under‐ or overprotection.^29^ To address such uncertainties, pesticides used in plant protection products in the UK require reassessment and reapproval every 15 years.^40^ However, no similar review process exists for veterinary medicines. The inclusion of an imidacloprid PNEC significantly higher than currently recognised thresholds in the phase II environmental risk assessment for Advocate Spot‐on Solution^22^, ^23^, ^24^ underscores the need for regular reassessment of environmental risks, particularly for intrinsically hazardous compounds such as parasiticides.

The interpretation of risk quotients within the VICH framework may also warrant reconsideration. Currently, a binary approach is applied, where a risk quotient of less than 1 indicates no risk, while greater than 1 signifies the presence of risk.^31^ However, adopting a staged risk ranking system (e.g., low, medium, high), as proposed elsewhere,^40^, ^41^, ^42^ could offer a more nuanced and realistic assessment. This is particularly relevant for parasiticides, which are often used on a large scale, have multiple potential environmental exposure pathways, and for which some degree of risk cannot be entirely excluded.

The samples in this study were collected starting 5 days post‐application, following datasheet guidelines that advise against swimming for 4 days after applying Advocate spot‐on. This sampling timeframe may have led to an underestimation of washoff emissions for fipronil‐based products, for which swimming is permitted from 3 days post‐application. Previous research has indicated a rapid decline in spot‐on emissions during the initial days following application, which the PEF estimated here may not reflect.^27^, ^43^

Our findings highlight gaps in the current regulatory framework and usage guidelines for spot‐on parasiticide products. Improving product labelling, restricting access (e.g., to prescription‐only status), raising consumer awareness and transitioning to risk‐based or reactionary parasite treatment can help to mitigate these emissions and promote more sustainable companion animal parasite control practices.

## AUTHOR CONTRIBUTIONS

Rosemary Perkins and Dave Goulson conceived and designed the study. Rosemary Perkins collected the data, analysed the data, prepared figures and tables and wrote the manuscript. Gaetan Glauser performed the chemical analysis and created the protocol for chemical extraction. All the authors were involved in manuscript writing.

## CONFLICT OF INTEREST STATEMENT

The authors declare that they have no known competing financial interests or personal relationships that could have appeared to influence the work reported in this paper.

## ETHICS STATEMENT

The study protocol was approved by the University of Sussex Animal Welfare and Ethical Review Body (reference ARG‐27/AWERB‐68), and an Animal Test Certificate Type‐S (reference 00733/2021) was issued by the Veterinary Medicines Directorate in accordance with Veterinary Medicines Regulations. Participating dog owners gave written consent prior to enrolment in the study.

## Supporting information



Supporting Information

## Data Availability

The data that support the findings of this study are available in the Supporting Information.
