# Supplementary material for: Swimming emissions from dogs treated with spot‐on fipronil or imidacloprid: Assessing the environmental risk
Source: Vet Rec. 2025 May 23;196(11):e5560. doi: 10.1002/vetr.5560 (PMC12124101; doi:10.1002/vetr.5560)
Supplement: Supplementary file 1 — Supporting Information [file VETR-196-e5560-s001.docx]

## Supplementary Information

### Dosage guidelines

*Table S1. Frontline spot-on for dogs dosage guidelines, by topical application to the skin according to bodyweight at a minimum of 6.7 mg/kg. (NOAH, 2021)*

| **Weight (kg)** | **Dosage (Pipettes)** | **Dosage (mg fipronil)** |
| --- | --- | --- |
| 2-10 | 1 × 0.67 ml pipette | 67 |
| >10 - 20 | 1 × 1.34 ml pipette | 134 |
| >20 - 40 | 1 × 2.68 ml pipette | 268 |
| >40-60 | 1 × 4.02 ml pipette | 402 |
| >60 | 2 × 2.68 pipettes | 534 |

*Table S2. Advocate spot-on for dogs dosage guidelines, by topical application to the skin according to bodyweight (NOAH, 2021) at a minimum 10 mg/kg* (1)

| **Weight (kg)** | **Dosage (Pipettes)** | **Dosage (mg imidacloprid)** |
| --- | --- | --- |
| ≤ 4 | 1 × 0.4ml pipette | 40 |
| >4 - 10 | 1 × 1.0 ml pipette | 100 |
| >10 - 25 | 1 × 2.5 ml pipette | 250 |
| >25 - 40 | 1 × 4.0 ml pipette | 400 |
| >40 | Appropriate combination of pipettes |  |

### Chemical analysis

Extraction was performed using a protocol adapted from Bonmatin *et al* (2019). Five hundred microlitres of sample were transferred to a 2.0 ml microcentrifuge tube, and 0.5 ml of chloroform as well as 12.5 µl of internal standard solution containing the 2 isotopically-labelled standards (imidacloprid-d_4_ at 4 µg/ml and fipronil-^13^C_3_ at 20 µg/ml in 50% methanol) were added to the tube. The tubes were vortexed for 10 s, hand-shaken for 20 s, and centrifuged for 5 min at 12000 *g*. Two hundred and fifty microlitres were collected from the lower organic phase and dried in a centrifugal evaporator at 35°C. Samples were reconstituted with 0.25 ml methanol 50%, diluted 100 times with methanol 50% and then transferred to an HPLC vial for analysis. The final concentration of isotopically-labelled internal standards were 1 ng/ml and 5 ng/ml for imidacloprid-d_4_ and fipronil-^13^C_3_, respectively.

All samples were analysed by ultra-high pressure liquid chromatography-tandem mass spectrometry (UHPLC-MS/MS) using a Waters Acquity UPLC I-Class coupled to a TQ-XS triple quadrupole mass spectrometer (Waters Corporation, Milford, MA, USA). An optimised method adapted from a well-established methodology by Kammoun *et al* (2019) was employed. Prior to the analysis of the experimental samples, we measured representative detergent-containing matrices spiked before extraction (equivalent to a final concentration of 1 ng/ml, n=3), which yielded recoveries of 104.4% and 105.3% for imidacloprid and fipronil, and precisions (expressed as %RSD) of 0.7 and 0.8%, respectively. The limit of detection (LOD) and lower limit of quantification (LLOQ) were defined as concentrations that gave peaks with signal-to-noise ratios of 3 and 10, respectively. LODs were 0.05 and 0.2 ng/mL and LLOQs were 0.2 and 0.8 ng/mL for imidacloprid and fipronil, respectively. For the analysis of the experimental samples, five batches of 70-120 samples were run over five consecutive days. For each batch, three conditioning samples were run, followed by the calibration curve (6 points at 0.005, 0.02, 0.1, 1, 5 and 10 ng/mL in 50% methanol, all containing imidacloprid-d_4_ and fipronil-^13^C_3_ at 1 and 5 ng/ml, respectively), three conditioning samples, two extracted blank water samples, three extracted blank water samples spiked at 1 ng/mL (used as quality controls) and the experimental samples. For every twenty samples, one of the quality control samples was run again to check for possible instrumental drifts.

### Derivation of Predicted No Effect Concentrations (PNECs)

**Imidacloprid**

1. **PNEC used in Phase II environmental risk assessment of Advocate Spot-on Solution®** (4)**:**

*‘The PNEC of 850 ug/l, for imidacloprid, is based on results from a 48-hour study on Daphnia magna which derived:*

- - *EC50 = 85 (range 71 – 113) mg/l (85 000 ug/l)*
  - *This EC50 was used together with a 100 fold assessment factor, yielding a PNEC of 850 ug/l. ‘*

**2. ECHA (European Chemicals Agency) PNEC for biocides** (5)

*‘The lowest effect value was obtained for the mayfly Caenis horaria (28d-EC10 = 0.024 µg/L). Normally, an assessment factor of 10 has to be applied, as long-term tests with species from 3 trophic levels are available. This would result in a PNEC_water_ of 0.0024 µg/L = 2.4 ng/L. However, for the derivation of the previous PNEC_water_ an assessment factor of 5 was used justified by the availability of a mesocosm study (Ratte et al., 2003) that shows that Chironomids were among the most sensitive species to imidacloprid. Although no statistical evaluation for Ephemeroptera could be performed in this study, it seems not appropriate to increase the assessment factor to 10 while the effect value for the PNEC derivation is significantly lower than the former effect value. Therefore, an assessment factor of 5 seems sufficiently conservative for the risk assessment. In addition there is an outdoor pond study availabe that show that Ephemeroptera are among the most sensitive taxa (Colombo et al, 2013). Thus, a reduction of the assessment factor to 5 would be possible as the uncertainty whether the most sensitive species/group is considered for the effects assessment is reduced. Therefore, it is proposed to apply an assessment factor of 5 on the lowest effect value found for Caenis horaria, resulting in a PNEC_water_ of 0.0048 µg/L = 4.8 ng/L.’*

**3. Norman database PNEC** (6)

Standardised methods to derive environmental quality standards are described by the NORMAN Network (7). The previous PNEC of 8.3 ng/l for imidacloprid was based on a species sensitivity distribution calculation (5,6). The calculation and data underlying the current PNEC of 6.8 ng/l are not clearly accessible on the NORMAN website – indicating a potential need for greater transparency on this database.

**Fipronil**

1. **ECHA (European Chemicals Agency) PNEC for biocides** (10)

*‘PNEC_surfacewater_ [12.1ng/L] was calculated from the lowest available freshwater NOEC (Chironomus riparius 28-d, NOEC = 0.121 µg a.s./L) with an Assessment Factor (AF) of 10 as long-term toxicity NOECs are available for at least three species representing three trophic levels’.*

1. **NORMAN database PNEC** (6)

The NORMAN database suggests a PNEC for fipronil of 0.77 ng/l. This was derived by applying an assessment factor of 10 to the 28-day NOEC (no observable effect concentration) of 7.7 ng/l for Mysidopsis bahia (5,6).

### Estimation of the Population Emission Fraction (PEF)

The PEF for swimming was estimated using the equation:

$$PEF= \sum_{d=n}^{d=28} {(WS}_{d}\times{PS}_{d})$$

Where *WS_d_* is the washoff percentage per swimming event on day *d* post-application, predicted from Box-cox transformed regression and *PS_d_* is the percentage of dogs that swam on day *d* post-application. n = 5 for imidacloprid and 3 for fipronil, based on datasheet guidelines. Input parameters are provided in Table S2. The assumption was made that instructions provided on the product label were followed, namely dogs treated with Advocate or Frontline spot-ons did not enter waterbodies before day 5 or day 3 following spot-on application, respectively (the day of application being day 1). Due to limited availability of data, the assumption was also made that emissions occurred for no more than one swimming event per treated animal and that no washoff occurred beyond 28 days post-application. Based on survey data, swimming frequency of spot-on treated dogs was taken as 17.9%, 7.1%, 11.2%, 5.8% and 6.2% of spot-on treated dogs swimming weekly, fortnightly, monthly, every 3 months or every 6 months (11).

*Table S3. Input parameters for estimation of population emission fractions through swimming for imidacloprid and fipronil swimming from dog spot-ons in the UK.*

| **Parameter** | **Nomenclature** | **Reference/Calculation** | **Comment** |
| --- | --- | --- | --- |
| Washoff percentage per swimming event on day *d* post-application | *WS_d_* | *Imidacloprid*:  *WS_d_* = {(1.9 -(*d**0.07))*λ + 1}^(1/λ)  λ = -0.06, R^2^ =0.43, p < 0.001  *Fipronil*:  *WS_d_* = {(1.24 -(*d**0.09)) *λ + 1}^(1/λ)  λ = 0.02, R^2^ =0.44, p < 0.001 |  |
| Percentage of dogs swimming on day *d* post spot-on application | *PS_d_* | ${PS}_{d} =\underset{d=5\to7}{\mathrm{Weekly}} \left( \frac{17.9}{3} \right)+\underset{d=5\to14}{\mathrm{Fortnightly}} \left( \frac{7.1}{10} \right)+\underset{d=5\to28}{\geq Monthly} \left( \frac{11.2}{24}+\frac{5.8}{87}+\frac{6.2}{178} \right)$ | Percentage values are drawn from Perkins and Goulson (2023). This equation assumes an even temporal distribution of swimming events – eg that dogs reported to swim fortnightly had an equal chance of swimming on any day between day 5 (when datasheet guidelines permit swimming) and day 14 post-application. |

### Results

*Table S4. Summary of swimming emissions from imidacloprid and fipronil spot-on treated dogs. Mean washoff with 95% confidence intervals at 5, 14 and 28 days post application expressed as % of applied mass, ranges expressed as % of applied mass and mg, n = number of samples.*

|  | **Mean washoff %** | | | | | | **Range**  **(%)** | **Range**  **(mg)** | **Total**  **Samples** |
| --- | --- | --- | --- | --- | --- | --- | --- | --- | --- |
|  | **Day 5** | **n** | **Day 14** | **n** | **Day 28** | **n** |  |  |  |
| **Fipronil** | 4.0 ± 1.9 | 8 | 0.8 ± 0.8 | 8 | 0.4 ± 0.2 | 8 | 0.1 - 7.7 | 0.2 - 8.6 | 24 |
| **Imidacloprid** | 10.1 ± 7.6 | 8 | 1.8 ± 0.8 | 9 | 1.4 ± 0.5 | 8 | 0.3 – 29.0 | 1.0 – 29 | 25 |

*Table S4. Mass washoff for fipronil and imidacloprid measured in swim water. Values are reported as mass and percentage washoff based on concentrations measured in representative sample and total volume of swim water. LOQ = limit of quantification. Active µg/l = measured concentration of applied active ingredient.*

| **Dog no** | **Active** | **Study day** | **Weight (kg)** | **Mg applied** | **Swim water (l)** | **Active µg/l** | **Imidacloprid**  **Washoff (mg)** | **Fipronil**  **Washoff (mg)** | **Imidacloprid**  **washoff %** | **Fipronil**  **washoff %** |
| --- | --- | --- | --- | --- | --- | --- | --- | --- | --- | --- |
| 1 | Imidacloprid | 5 | 8.9 | 100 | 204.5 | 92.34 | 18.88 | <LOQ | 18.88 | na |
| 2 | Imidacloprid | 5 | 27.5 | 400 | 296 | 64.09 | 18.97 | 0.36 | 4.74 | na |
| 3 | Imidacloprid | 5 | 24.8 | 250 | 378.1 | 45.47 | 17.19 | <LOQ | 6.88 | na |
| 4 | Imidacloprid | 5 | 6.4 | 100 | 176 | 50.83 | 8.95 | <LOQ | 8.95 | na |
| 5 | Imidacloprid | 5 | 15.2 | 250 | 254 | 38.34 | 9.74 | <LOQ | 3.90 | na |
| 6 | Imidacloprid | 5 | 13 | 250 | 246 | 51.64 | 12.70 | 0.36 | 5.08 | na |
| 7 | Imidacloprid | 5 | 5.2 | 100 | 67 | 432.4 | 28.97 | <LOQ | 28.97 | na |
| 8 | Imidacloprid | 5 | 10.1 | 250 | 240 | 35 | 8.40 | <LOQ | 3.36 | na |
| 9 | Imidacloprid | 14 | 29.7 | 400 | 320.5 | 3.26 | 1.04 | <LOQ | 0.26 | na |
| 10 | Imidacloprid | 14 | 19.3 | 250 | 319.9 | 25.12 | 8.04 | <LOQ | 3.21 | na |
| 11 | Imidacloprid | 14 | 4.5 | 100 | 66.5 | 26.06 | 1.73 | <LOQ | 1.73 | na |
| 12 | Imidacloprid | 14 | 22.5 | 250 | 249.2 | 26.54 | 6.61 | <LOQ | 2.65 | na |
| 13 | Imidacloprid | 14 | 11.2 | 250 | 196 | 17.28 | 3.39 | <LOQ | 1.35 | na |
| 14 | Imidacloprid | 14 | 11.2 | 250 | 214 | 19.41 | 4.15 | <LOQ | 1.66 | na |
| 15 | Imidacloprid | 14 | 29 | 400 | 319 | 12.71 | 4.05 | <LOQ | 1.01 | na |
| 16 | Imidacloprid | 14 | 7.2 | 100 | 61 | 40.94 | 2.50 | <LOQ | 2.50 | na |
| 17 | Imidacloprid | 28 | 23.3 | 250 | 353.5 | 17.13 | 6.06 | <LOQ | 2.42 | na |
| 18 | Imidacloprid | 28 | 8.3 | 100 | 64.1 | 32.35 | 2.07 | <LOQ | 2.07 | na |
| 19 | Imidacloprid | 28 | 12.6 | 250 | 194.8 | 14.49 | 2.82 | <LOQ | 1.13 | na |
| 20 | Imidacloprid | 28 | 26.5 | 400 | 332 | 7.41 | 2.46 | <LOQ | 0.62 | na |
| 21 | Imidacloprid | 28 | 6.4 | 100 | 38 | 39.8 | 1.51 | <LOQ | 1.51 | na |
| 22 | Imidacloprid | 28 | 12 | 250 | 250 | 10.5 | 2.63 | <LOQ | 1.05 | na |
| 23 | Imidacloprid | 28 | 11 | 250 | 256 | 12.43 | 3.18 | <LOQ | 1.27 | na |
| 24 | Imidacloprid | 28 | 17.6 | 250 | 289 | 8.29 | 2.40 | <LOQ | 0.96 | na |
| 25 | Fipronil | 5 | 10.5 | 134 | 190 | 26.48 | <LOQ | 5.03 | na | 3.75 |
| 26 | Fipronil | 5 | 10.5 | 134 | 212 | 28.67 | <LOQ | 6.08 | na | 4.54 |
| 27 | Fipronil | 5 | 17 | 134 | 201 | 33.04 | 0.08 | 6.64 | na | 4.96 |
| 28 | Fipronil | 5 | 14 | 134 | 390 | 4.36 | <LOQ | 1.70 | na | 1.27 |
| 29 | Fipronil | 5 | 8.1 | 67 | 144 | 35.84 | <LOQ | 5.16 | na | 7.70 |
| 30 | Fipronil | 5 | 14 | 134 | 305.5 | 27.25 | <LOQ | 8.32 | na | 6.21 |
| 31 | Fipronil | 5 | 9.7 | 67 | 226 | 5.01 | <LOQ | 1.13 | na | 1.69 |
| 32 | Fipronil | 5 | 24.8 | 268 | 326 | 17.66 | <LOQ | 5.76 | na | 2.15 |
| 33 | Fipronil | 14 | 9.2 | 67 | 59 | 2.71 | <LOQ | 0.16 | na | 0.24 |
| 34 | Fipronil | 14 | 13.8 | 134 | 210 | 5.3 | <LOQ | 1.11 | na | 0.83 |
| 35 | Fipronil | 14 | 10.7 | 134 | 398 | 11.6 | <LOQ | 4.62 | na | 3.45 |
| 36 | Fipronil | 14 | 32.2 | 268 | 453 | 1.0 | <LOQ | 0.45 | na | 0.17 |
| 37 | Fipronil | 14 | 8 | 67 | 241 | 1.11 | <LOQ | 0.27 | na | 0.40 |
| 38 | Fipronil | 14 | 10.7 | 134 | 283 | 1.84 | <LOQ | 0.52 | na | 0.39 |
| 39 | Fipronil | 14 | 27.3 | 268 | 396 | 3.78 | <LOQ | 1.50 | na | 0.56 |
| 40 | Fipronil | 14 | 33 | 268 | 380 | 0.55 | <LOQ | 0.21 | na | 0.08 |
| 41 | Fipronil | 14 | 9.5 | 67 | 170 | 4.6 | <LOQ | 0.78 | na | 1.17 |
| 42 | Fipronil | 28 | 15 | 134 | 316 | 0.93 | <LOQ | 0.29 | na | 0.22 |
| 43 | Fipronil | 28 | 9.8 | 67 | 261 | 1.33 | <LOQ | 0.35 | na | 0.52 |
| 44 | Fipronil | 28 | 24.5 | 268 | 388 | 1.35 | <LOQ | 0.52 | na | 0.20 |
| 45 | Fipronil | 28 | 15.7 | 134 | 310 | 1.26 | <LOQ | 0.39 | na | 0.29 |
| 46 | Fipronil | 28 | 13.3 | 134 | 243.8 | 2.77 | 0.36 | 0.68 | na | 0.50 |
| 47 | Fipronil | 28 | 8.4 | 67 | 156 | 1.68 | <LOQ | 0.26 | na | 0.39 |
| 48 | Fipronil | 28 | 10.7 | 134 | 226 | 0.93 | 0.04 | 0.21 | na | 0.16 |
| 49 | Fipronil | 28 | 31 | 268 | 414.1 | 5.01 | <LOQ | 2.07 | na | 0.77 |

### References

1. Marsella R. Advances in flea control. Vet Clin North Am - Small Anim Pract. 1999;

2. Bonmatin JM, Noome DA, Moreno H, Mitchell EAD, Glauser G, Soumana OS, et al. A survey and risk assessment of neonicotinoids in water, soil and sediments of Belize. Environ Pollut. 2019;249:949–58.

3. Kammoun S, Mulhauser B, Aebi A, Mitchell EAD, Glauser G. Ultra-trace level determination of neonicotinoids in honey as a tool for assessing environmental contamination. Environ Pollut. 2019;247:964–72.

4. VMD (Veterinary Medicines Directorate). Freedom of Information Request ATI0731. 2021.

5. ECHA (European Chemicals Agency). Imidacloprid Product-type 18 18th Directive 98/8/EC concerning the placing of biocidal products on the market [Internet]. 2015. Available from: https://echa.europa.eu/documents/10162/225b9c58-e24c-6491-cc8d-7d85564f3912

6. NORMAN. NORMAN Ecotoxicology database. 2024; Available from: https://www.norman-network.com/nds/ecotox/. Accessed November 2024

7. NORMAN. Deriving Environmental Quality Standards for chemical substances in surface waters [Internet]. 2024 [cited 2024 Feb 4]. Available from: https://www.norman-network.com/nds/ecotox/docs/Fact-sheet-EQS-Derivation.pdf

8. NORMAN Ecotoxicology Database [Internet]. 2019 [cited 2019 Dec 18]. Available from: https://www.norman-network.com/nds/ecotox/lowestPnecsIndex.php

9. Perkins R, Whitehead M, Civil W, Goulson D. Potential Role of Veterinary Flea Products in Widespread Pesticide Contamination of English Rivers. Sci Total Environ [Internet]. 2021;750(1):143560. Available from: https://doi.org/10.1016/j.scitotenv.2020.143560

10. ECHA (European Chemicals Agency). Fipronil Product-type PT18. Directive 98/8/EC concerning the placing biocidal products on the market [Internet]. 2011. Available from: http://dissemination.echa.europa.eu/Biocides/ActiveSubstances/0033-18/0033-18_Assessment_Report.pdf

11. Perkins R, Goulson D. To flea or not to flea: Survey of UK companion animal ectoparasiticide usage and activities affecting pathways to the environment. PeerJ. 2023;11:15561.
